# Different Perivascular Space Burdens in Idiopathic Rapid Eye Movement Sleep Behavior Disorder and Parkinson’s Disease

**DOI:** 10.3389/fnagi.2020.580853

**Published:** 2020-11-05

**Authors:** Xiao-li Si, Lu-yan Gu, Zhe Song, Cheng Zhou, Yi Fang, Chong-yao Jin, Jing-jing Wu, Ting Gao, Tao Guo, Xiao-jun Guan, Xiao-jun Xu, Xin-zhen Yin, Ya-ping Yan, Min-min Zhang, Jia-li Pu

**Affiliations:** ^1^Department of Neurology, The Second Affiliated Hospital, College of Medicine, Zhejiang University, Hangzhou, China; ^2^Department of Radiology, The Second Affiliated Hospital, College of Medicine, Zhejiang University, Hangzhou, China

**Keywords:** Parkinson’s disease, REM sleep behavior disorder, perivascular space, glymphatic system, white matter hyperintensity

## Abstract

**Background:**

Excessive aggregation of α-synuclein is the key pathophysiological feature of Parkinson’s disease (PD). Rapid eye movement sleep behavior disorder (RBD) is also associated with synucleinopathies and considered as a powerful predictor of PD. Growing evidence suggests the diminished clearance of α-synuclein may be partly attributable to poor interstitial fluid drainage, which can be reflected by magnetic resonance imaging (MRI)-visible enlarged perivascular space (EPVS). However, the effect of MRI-visible EPVS on iRBD and PD, and their correlation with clinical characteristics remain unclear.

**Objective:**

To evaluate the clinical and neuroimaging significance of MRI-visible EPVS in iRBD and PD patients.

**Methods:**

We enrolled 33 iRBD patients, 82 PD (with and without RBD) patients, and 35 healthy controls (HCs), who underwent clinical evaluation and 3.0 Tesla MRI. Two neurologists assessed MRI-visible EPVS in centrum semiovale (CSO), basal ganglia (BG), substantia nigra (SN), and brainstem (BS). Independent risk factors for iRBD and PD were investigated using multivariable logistic regression analysis. Spearman analysis was used to test the correlation of MRI-visible EPVS with clinical characteristics of patients.

**Results:**

iRBD patients had significantly higher EPVS burdens (CSO, BG, SN, and BS) than PD patients. Higher CSO-EPVS and BS-EPVS burdens were independent risk factors for iRBD. Furthermore, higher CSO-EPVS and SN-EPVS burdens were positively correlated with the severity of clinical symptom in iRBD patients, and higher BG-EPVS burden was positively correlated with the severity of cognitive impairment in PD patients.

**Conclusion:**

iRBD and PD patients have different MRI-visible EPVS burdens, which may be related with a compensatory mechanism in glymphatic system. Lower MRI-visible EPVS burden in PD patients may be a manifestation of severe brain waste drainage dysfunction. These findings shed light on the pathophysiologic relationship between iRBD and PD with respect to neuroimaging marker of PD.

## Introduction

Parkinson’s disease (PD) is an age-related neurodegenerative disorder that is caused by the loss of dopaminergic (DA) neurons in the substantia nigra (SN) and is characterized by misfolded α-synuclein in Lewy bodies (Sulzer and Surmeier, 2013). Apart from classical motor symptoms, PD is also associated with a broad spectrum of non-motor symptoms, including autonomic dysfunction, sensory symptoms, neuropsychiatric dysfunction and sleep disturbances (Poewe, 2008; Xie et al., 2019; LeWitt et al., 2020).

Before the appearance of PD classical motor symptoms, at least 60% DA neurons have degenerated (Si et al., 2017). Therefore, detecting a premotor marker which aids in primary prevention of PD is in urgent need. Rapid eye movement (REM) sleep behavior disorder (RBD), which is characterized by recurrent nocturnal dream enactment behavior during REM sleep without normal muscle atonia, commonly accompanies PD and even precede the onset of PD (Arnulf, 2012). A growing number of studies have focused on idiopathic RBD (iRBD) to be an prodromal marker of PD (Schenck et al., 2013; Xie et al., 2019; Zhang et al., 2020). iRBD is strongly associated with synucleinopathy and there is a long time interval (∼10 years) between the onset of iRBD and PD, which provides the time window for disease-modifying therapies (Xie et al., 2019). However, it is challenging to make assumptions about the phenoconversion and disease subtypes that may subsequently follow iRBD (Qian and Huang, 2019). As imaging is less susceptible to subjectivity and drug influence, the establishment of neuroimaging markers to detect brain changes in patients with iRBD and PD is promising, and may be a valuable method for predicting PD disease progression.

Glymphatic system, the lymphatic drainage system of central nervous system (CNS), may contribute to the clearance of α-synuclein (Jessen et al., 2015; Li et al., 2020). Perivascular spaces (PVSs), also known as Virchow-Robin spaces, form a network of spaces around microvessels that are dedicated to cerebrospinal fluid (CSF) transportation and metabolic waste drainage, are major component of the glymphatic system (Iliff et al., 2012; Jessen et al., 2015; Mestre et al., 2017). However, normal PVSs are invisible on conventional structural magnetic resonance image (MRI) and can only be visualized when enlarged (Potter et al., 2015). Enlarged PVSs (EPVSs) are spaces with diameters less than 3 mm, which are visible on MRI. MRI-visible EPVSs are commonly found in centrum semiovale (CSO), basal ganglia (BG), and brainstem (BS), with high signals on T2-weighted images and low signals on T1-weighted and fluid attenuated inversion recovery (FLAIR) images (Wardlaw et al., 2013b). MRI-visible EPVS burden is a cerebral small vessel disease (SVD) and is predictive of cognitive impairment in cerebrovascular disease (Halliday et al., 2014; Gutierrez et al., 2017). Yet it likely resembles obstruction by protein and stagnation of fluid (Brown et al., 2018), which may contribute to the development of neurodegenerative diseases (Banerjee et al., 2017), including PD and iRBD (Laitinen et al., 2000; Xie et al., 2013; Park Y. W. et al., 2019). However, the effect of MRI-visible EPVSs in iRBD and PD patients, and their correlation with clinical characteristics remains unclear.

Therefore, by evaluating MRI-visible EPVS burdens in CSO, BG, SN and BS, we investigated the clinical significance of MRI-visible EPVS in iRBD and PD patients. To the best of our knowledge, this is the first study to investigate the relationship between these two closely linked diseases in terms of neuroimaging-based markers, which may shed light on the pathophysiology of iRBD and PD.

## Materials and Methods

### Study Design and Participants

This study was approved by the ethics committee of the Second Affiliated Hospital of the Zhejiang University School of Medicine, and written consent was obtained from each participant. The participants were selected from the outpatient clinic of the Department of Neurology of the Second Affiliated Hospital of the Zhejiang University School of Medicine for adjacent communities, between January 2017 and August 2019. Patients who met the American Academy of Sleep Medicine criteria for iRBD were assigned to the iRBD group (American Academy of Sleep Medicine, 2014), while patients who met the Movement Disorder Society’s Clinical Diagnostic Criteria for PD were assigned to the PD group (Postuma et al., 2015). The criteria of classification of PD with symptomatic RBD (PD-sRBD) group and PD without RBD (PD-nRBD) group was according to REM Sleep Behavior Disorder Questionnaire-Hong Kong (RBDQ-HK) scale (Shen et al., 2014). Age- and sex-matched community volunteers served as healthy controls (HCs). Potential participants who met the following exclusion criteria were excluded: (1) sleep-related epilepsy, obstructive sleep apnea hypopnea syndrome, night terror/sleepwalking, nocturnal paroxysmal dystonia, or secondary RBD caused by drugs; (2) other neurodegenerative diseases, such as dementia with Lewy bodies (DLB), multiple system atrophy (MSA), progressive supranuclear palsy, or secondary PD caused by drugs; (3) experience of a cerebrovascular accident, such as cerebral hemorrhage, cerebral infarction, subarachnoid hemorrhage, etc.; (4) cerebral structural abnormality, such as brain trauma and brain tumor; (5) have severe cognitive dysfunction [Montreal Cognitive Assessment (MoCA) score < 10 and/or Mini-Mental State Examination (MMSE) score <9] (Dalrymple-Alford et al., 2010; Arevalo-Rodriguez et al., 2015); (6) inability to undergo MRI due to contraindications, such as metal implants and mental disorders; and (7) refusal to sign the informed consent form. In total, 35 age- and sex- matched HCs, 33 patients with iRBD, and 82 patients with PD (43 PD-nRBD and 39 PD-sRBD patients) were enrolled. All the participants underwent clinical evaluation and 3.0 Tesla (3.0T) MRI scan.

### Clinical Assessment

We performed clinical evaluation of all the participants, including detailed personal information, medical history, and neurological and neuropsychological examination. The severity and stage of PD were evaluated with the Unified Parkinson’s Disease Rating Scale (UPDRS) and the Hoehn-Yahr scale (H-Y stage), respectively. The RBDQ-HK scale was used to evaluate the severity of iRBD patients and classify the PD-sRBD and PD-nRBD groups (using a cut-off score of 17/18 points for the overall scale and 7/8 points for the factor II subscale of the RBDQ-HK) (Shen et al., 2014). In addition, we performed the MMSE and MoCA scales to evaluate cognitive function. The participants’ demographic and clinical characteristics are summarized in Table 1.

**TABLE 1:** Baseline characteristics and Imaging findings according to disease classification.

	**HC**	**iRBD**	**PD**		***P*-value**	
			**PD-nRBD**	**PD-sRBD**	**HC vs. RBD vs. PD**	**HC vs. RBD vs. PD-nRBD vs. PD-sRBD**
n (%)	35 (23.2)	33 (21.9)	43 (28.5)	39 (27.9)	–	–
**Clinical variables**						
Age, years, mean (SD)	61.3 (7.0)	65.6 (8.9)	59.2 (12.1)	61.8 (8.3)	0.056	0.109
Sex, male, n (%)	12 (34.3)	17 (51.5)	21 (48.8)	26 (66.7)	0.075	0.051
Smoking, n (%)	5 (14.3)	10 (30.3)	7 (16.3)	12 (30.8)	0.283	0.118
Hypertension, n (%)	11 (31.4)	8 (24.2)	13 (30.2)	9 (23.1)	0.617	0.686
Hyperlipidaemia, n (%)	4 (11.4)	4 (12.1)	2 (4.7)	1 (2.6)	0.167	0.294
Diabetes mellitus, n (%)	1 (2.9)	2 (6.1)	1 (2.3)	4 (10.3)	0.759	0.379
MMSE, mean (SD)	27.5 (2.5)	27.2 (3.0)	26.5 (4.6)	26.3 (3.9)	0.589	0.675
MoCA, mean (SD)	24.3 (3.4)	22.6 (4.5)	21.5 (6.2)	20.9 (5.4)	**0.022**	**0.034**
RBDQ-HK, mean (SD)	7.4 (4.7)	39.4 (12.9)	7.5 (4.8)	27.1 (13.4)	**0.000**	**0.000**
RBDQ-HK II, mean (SD)	0.9 (1.7)	24.5 (10.9)	2.1 (2.4)	16.5 (10.5)	**0.000**	**0.000**
Disease duration, years, mean (SD)	–	–	3.8 (3.7)	4.6 (5.3)	–	0.346*
UPDRS total, mean (SD)	–	–	30.6 (19.2)	31.2 (18.3)	–	0.446*
UPDRS I	–	–	1.5 (1.6)	1.5 (2.0)	–	0.965*
UPDRS II	–	–	8.3 (5.7)	8.8 (5.4)	–	0.530*
UPDRS III	–	–	19.9 (13.6)	20.3 (12.5)	–	0.492*
UPDRS IV	–	–	0.9 (1.7)	0.8 (1.1)	–	0.675*
H-Y stage, mean (SD)	–	–	2.2 (0.9)	2.0 (0.6)	–	0.435*
**Imaging findings**						
CSO-EPVS, mean (SD)	17.0 (7.9)	21.3 (8.5)	16.3 (10.4)	17.1 (8.6)	**0.019**	**0.034**
BG-EPVS, mean (SD)	12.4 (3.7)	15.0 (5.4)	11.0 (6.2)	12.1 (7.3)	**0.001**	**0.002**
SN-EPVS, mean (SD)	1.9 (1.1)	2.7 (1.7)	1.8 (1.3)	1.5 (1.1)	**0.024**	**0.038**
BS-EPVS, mean (SD)	2.3 (1.5)	3.3 (1.6)	2.6 (1.6)	2.2 (1.4)	**0.020**	**0.023**
WMH score, mean (SD)	2.3 (1.6)	2.4 (1.4)	1.9 (1.4)	1.8 (1.3)	0.056	0.122

MMSE, Mini-mental State Examination; MoCA, Montreal Cognitive Assessment; RBDQ-HK, REM Sleep Behavior Disorder Questionnaire-Hong Kong; UPDRS, United Parkinson’s Disease Rate Scale; H-Y stage, Hoehn-Yahr stage; EPVS, enlarged perivascular space; CSO, centrum semiovale; BG, basal ganglia; SN,substantia nigra; BS, brainstem; WMH, white matter hyperintensity. *P-values reflect comparisons between PD-nRBD and PD-sRBD groups using Mann-Whitney *U*-tests. P-value < 0.05.

### MRI Acquisition

Standardized T2 and FLAIR images were acquired from all the participants at the Department of Radiology of the Second Affiliated Hospital of the Zhejiang University School of Medicine, using the same 3.0 Tesla MRI scanner (GE Discovery 750). The images for each participant were obtained in one session, and all the MRIs were obtained in the same orientation and slice positions. We acquired the T2 gradient recalled echo (GRE) images with the following imaging parameters: repetition time (TR) = 3,000 ms, echo time (TE) = 102 ms, flip angle = 90°, field of view = 240 mm× 240 mm, matrix size = 256 × 256 pixels, and slice number/thickness = 38/4.0 mm. FLAIR GRE-images were obtained using the following parameters: TR = 11,000 ms, TE = 147 ms, flip angle = 90°, field of view = 220 mm× 220 mm, matrix size = 256 × 256 pixels, and slice number/thickness = 42/4.0 mm.

### Quantification of MRI-Visible EPVS

**FIGURE 1:**
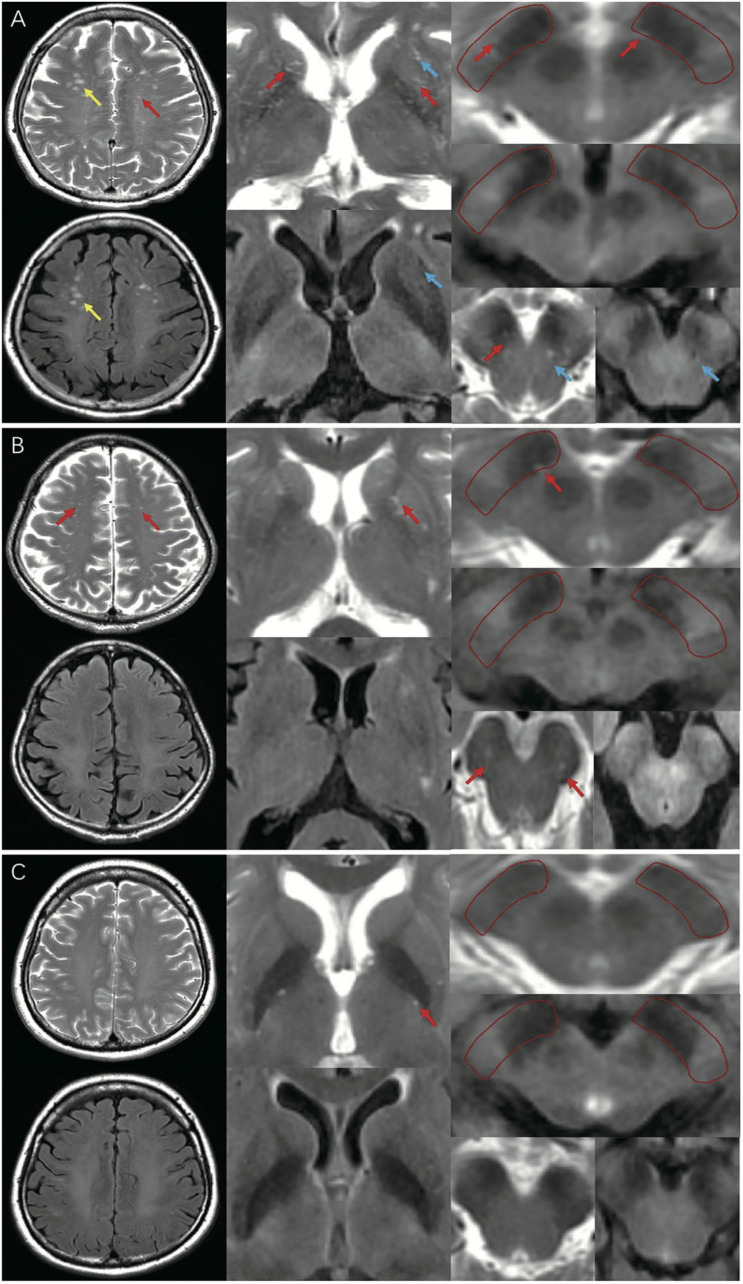
MRI-visible EPVS on T2 and FLAIR imaging. Red arrows indicate EPVS: an EPVS was defined as a fluid-filled space from 1 to 3 mm in diameter with high signal on T2 and low signal on FLAIR imaging, without a perilesional halo. Yellow arrows indicate WMH: a WMH was defined as a lesion with variable size in the white matter, with high signal on both T2 and FLAIR imaging. Blue arrows indicate lacunes: a lacune was defined as a lesion 3–15 mm in diameter with high signal on T2 and a perilesional halo on FLAIR imaging. **(A)** A representative of EPVS in iRBD group (frequent/severe grade CSO, BG-EPVS, with EPVS in SN and BS); **(B)** A representative of EPVS in HC group (moderate grade CSO, BG-EPVS, with EPVS in SN and BS); **(C)** A representative of EPVS in PD group (none/mild grade CSO, BG-EPVS, without EPVS in SN or BS). EPVS, enlarged perivascular spaces; FLAIR: fluid attenuated inversion recovery; WMH, white matter hyperintensity; CSO, centrum semiovale; BG, basal ganglia; SN, substantia nigra; BS, brainstem; MRI, magnetic resonance imaging.

MRI-visible EPVS was defined as a fluid-filled space (1–3 mm in diameter) that followed the typical course of a vessel, whose space had a signal intensity similar to CSF on all sequences. Evaluation was done by two trained neurologists (S.XL and G.LY), according to the STandards for ReportIng Vascular changes on nEuroimaging (STRIVE) criteria (Wardlaw et al., 2013a; Hilal et al., 2018b; Figure 1). These two neurologists were blinded to the clinical data. Discrepancies were resolved by negotiation. The numbers of MRI-visible EPVS in both hemispheres were assessed and added together. We selected the slices of CSO, BG, and BS with the highest numbers of EPVS after scanning all the relevant slices. We also selected the specific region of SN that we have marked in Figure 1A. The inter-rater reliability was good for BG-EPVS (Spearman’s rank correlation coefficient, intraclass correlation coefficient [ICC] = 0.58) and CSO-EPVS (ICC = 0.46), and excellent for SN-EPVS (ICC = 0.88) and BS-EPVS (ICC = 0.89), respectively.

### Measurement of White Matter Hyperintensity

White matter hyperintensities (WMHs) were defined as signal abnormalities of variable sizes in the white matter, which exhibited hyperintensity on T2-weighted images, such as FLAIR, without cavitation (that is, the signal is different from that of CSF) according to STRIVE criteria (Wardlaw et al., 2013a; Figure 1). We used the Fazekas scale to grade periventricular (PV) WMHs (PVWMHs) and deep WMHs (DWMHs). PVWMH scores were 0 = absent, 1 = caps or pencil-thin lining, 2 = smooth halo, and 3 = irregular PV lesions extending into the deep white matter. DWMH scores were 0 = absent, 1 = punctate foci, 2 = beginning confluence of foci, and 3 = large confluent areas (Fazekas et al., 1987). The total WMH (Fazekas) score was calculated by summing the scores of these two regions, and a total score >3 was defined as severe WMH (Park et al., 2019). WMHs were rated by two trained neurologists (S.Z and Z.C). Discrepancies were resolved by negotiation.

### Statistical Analysis

Data were entered into Microsoft Excel, and statistical analyses were performed with Statistical Package for the Social Sciences 18.0 (IBM, Chicago, IL, United States). Statistical plots were generated using GraphPad Prism 7.0a (GraphPad Inc., San Diego, CA, United States). The results are expressed as mean ± standard deviation (mean ± SD) for continuous variables and as frequencies for categorical variables. The Kolmogorov-Smirnov test was used to determine the normality of the distribution of the variables. Demographic data, clinical variables, and MRI-visible EPVS were compared using independent *t*-tests for normally distributed continuous variables, and Mann-Whitney *U* test and Kruskal-Wallis test for continuous variables that were not normally distributed. Kruskal-Wallis Multiple Compare (Nemenyi test) was used for multiple comparisons, and a corrected *P* < 0.05 was considered statistically significant. We performed both univariate and multivariate logistic regression analyses. Variables of interest in the univariate analysis (*P* < 0.05) and previously known demographic and clinical risk factors, including age, sex, smoking, hypertension, hyperlipidemia, diabetes mellitus, MoCA score, MRI-visible EPVS, and WMH score, were included in the multivariable models by using backward elimination according to the likelihood ratio, with a variable selection criterion of *P* < 0.05. We estimated the area under the receiver operating characteristic curve (AUC) to assess the predictive ability of the multivariable models. Spearman’s rank correlation was used to evaluate the bivariate associations between number of EPVS and RBDQ-HK (total and subscale II) scores, UPDRS (total and III) scores, and MoCA scores in HC, iRBD, and PD groups. *P* < 0.05 was regarded as statistically significant.

## Results

### Sample and Participant Characteristics

In total, 239 participants completed the clinical data collection and underwent 3.0T MRI. Based on the inclusion and exclusion criteria, the data of 33 iRBD patients, 82 PD patients (43 PD-nRBD and 39 PD-sRBD patients), and 35 HCs were analyzed for the study. The demographic and clinical characteristics of the participants are summarized in Table 1. There was no significant difference among the four groups (*P* > 0.05 with Kruskal-Wallis test) with respect to baseline age, sex, smoking, hypertension, hyperlipidemia, diabetes mellitus, or MMSE scores. The PD-sRBD group had lower MoCA scores (20.9 ± 5.4 vs. 24.3 ± 3.4, *P* = 0.023) than HC group. The iRBD and PD-sRBD groups had higher RBDQ-HK (total and II) scores (*P* < 0.05) than the HC and PD-nRBD groups. A subgroup analysis between PD-nRBD group and PD-sRBD group found no significant difference in demographic or clinical characteristics of patients [including disease duration, UPDRS scores (I, II, III, IV, and total), and Hoehn-Yahr stage] in these two groups (*P* > 0.05).

### Distribution of EPVSs

**FIGURE 2:**
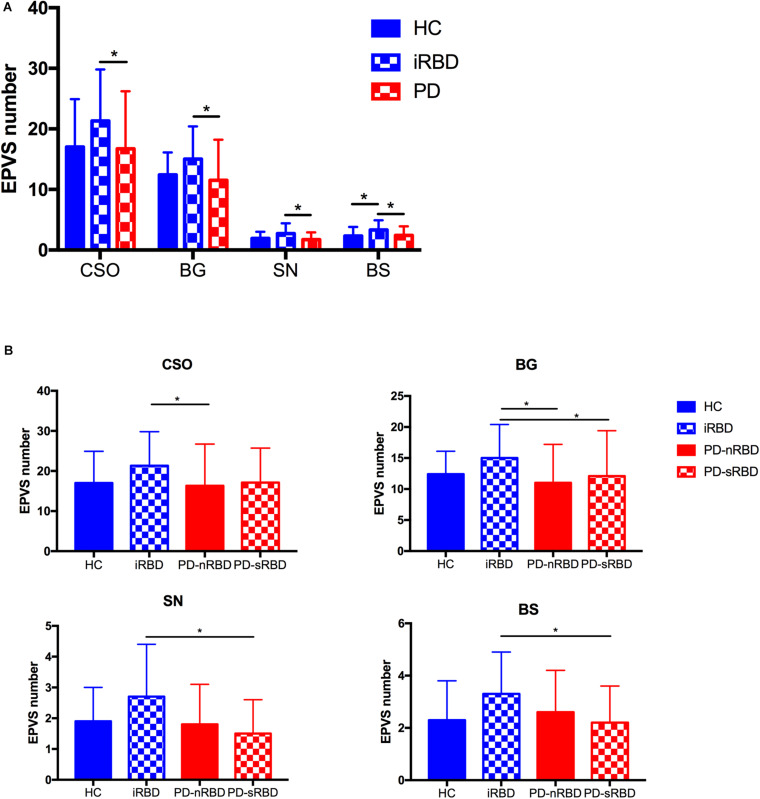
**(A)** Comparison of numbers of EPVS among HC, iRBD, and PD (with or without RBD) groups; **(B)** Comparison of numbers of CSO-EPVS, BG-EPVS, SN-EPVS, and BS-EPVS (mean ± SD) among HC, iRBD, PD-nRBD, and PD-sRBD groups. *P < 0.05. HC, healthy control; iRBD, idiopathic rapid eye movement sleep behavior disorder; PD, Parkinson’s disease; PD-nRBD, Parkinson’s disease without symptomatic RBD; PD-sRBD, Parkinson’s disease with symptomatic RBD; EPVS, enlarged perivascular space; CSO, centrum semiovale; BG, basal ganglia; SN, substantia nigra; BS, brainstem; SD, standard deviation.

The imaging characteristics (numbers of EPVS and WMH scores) of the four groups are summarized in Table 1 and Figure 2. Kruskal-Wallis Multiple Compare was used for multiple comparisons. The subgroup analysis between HC and iRBD groups showed that iRBD group had higher numbers of CSO-, BG-, and SN-EPVS burdens (*P* > 0.05), and higher BS-EPVSs (3.3 ± 1.6 vs. 2.3 ± 1.5, *P* = 0.032) than HC group. The subgroup analysis between iRBD and PD groups showed that iRBD group had significantly higher numbers of CSO-EPVS (21.3 ± 8.5 vs. 16.7 ± 9.5, *P* = 0.015), BG-EPVS (15.0 ± 5.4 vs. 11.5 ± 6.7, *P* = 0.001), SN-EPVS (2.7 ± 1.7 vs. 1.7 ± 1.2, *P* = 0.020), and BS-EPVS (3.3 ± 1.6 vs. 2.4 ± 1.5, *P* = 0.044) than PD group. There was no significant difference in numbers of EPVS between PD and HC groups (*P* > 0.05). There was no significant difference of WMH scores in these four groups (*P* > 0.05) (Table 1 and Figure 2A). Among PD patients, the iRBD group had significant higher numbers of CSO-EPVS (21.3 ± 8.5 vs. 16.3 ± 10.4, *P* = 0.022) and BG-EPVS (15.0 ± 5.4 vs. 11.0 ± 6.2, *P* = 0.003) than PD-nRBD group, while iRBD group had significantly higher numbers of BG-EPVS (15.0 ± 5.4 vs. 12.1 ± 7.3, *P* = 0.010), SN-EPVS (2.7 ± 1.7 vs. 1.5 ± 1.1, *P* = 0.025), and BS-EPVS (3.3 ± 1.6 vs. 2.2 ± 1.4, *P* = 0.034) than PD-sRBD group. There was no significant difference in numbers of EPVS between PD-nRBD and PD-sRBD groups (Figure 2B).

### Logistic Regression Results

The univariate analysis of the HC and iRBD groups showed that higher numbers of CSO-EPVS (OR = 1.07; 95% CI: 1.00–1.14), BG-EPVS (OR = 1.13; 95% CI: 1.01–1.27), SN-EPVS (OR = 1.47; 95% CI: 1.02–2.12), and BS-EPVS (OR = 1.50; 95% CI: 1.06–2.13) were associated with the diagnosis of iRBD (*P* < 0.05). In multivariable analysis, lower MoCA score (OR = 0.79; 95% CI: 0.66–0.94, P = 0.008), and higher numbers of CSO-EPVS (OR = 1.10; 95% CI: 1.00–1.21, P = 0.048) and BS-EPVS (OR = 1.95; 95% CI = 1.18–3.23, *P* = 0.010) were independent risk factors for iRBD (Table 2). The model achieved an AUC of 0.68 (95% CI: 0.55–0.81).

**TABLE 2:** Logistic regression analysis for clinical and imaging predictors of iRBD.

**Variables**	**Univariate**		**Multivariable**	
	**OR (95% CI)**	***P*-value**	**OR (95% CI)**	***P*-value**
**Clinical variables**				
Age, years	1.05 (0.99–1.12)	0.082	–	–
Sex, male	0.49 (0.19-1.30)	0.153	–	–
Smoking	2.25 (0.67–7.61)	0.192	–	–
Hypertension	1.63 (0.57–4.70)	0.365	–	–
Hyperlipidaemia	1.29 (0.27–6.26)	0.752	–	–
Diabetes mellitus	0.29 (0.03–2.98)	0.300	–	–
MoCA	0.89 (0.78–1.01)	0.080	0.79 (0.66–0.94)	**0.008**
**Imaging findings**				
CSO-EPVS	1.07 (1.00–1.14)	**0.041**	1.10 (1.00–1.21)	**0.048**
BG-EPVS	1.13 (1.01–1.27)	**0.030**	–	–
SN-EPVS	1.47 (1.02–2.12)	**0.037**	–	–
BS-EPVS	1.50 (1.06–2.13)	**0.022**	1.95 (1.18–3.23)	**0.010**
WMH score	1.05 (0.77–1.44)	0.760	–	–

*OR, odds ratio; MoCA, Montreal Cognitive Assessment; EPVS, enlarged perivascular spaces; CSO, centrum semiovale; BG, basal ganglia; SN, substantia nigra; BS, brainstem; WMH, white matter hyperintensity. P-value < 0.05.*

The univariate analysis of the entire sample showed that lower MoCA score (OR = 0.91; 95% CI: 0.85–0.98), fewer BG-EPVS (OR = 0.94; 95% CI: 0.89–0.99) and SN-EPVS (OR = 0.71; 95% CI: 0.55–0.92), and lower WMH score (OR = 0.76; 95% CI: 0.60–0.95) were associated with the diagnosis of PD. The multivariate analysis found that female sex (OR = 0.33; 95% CI = 0.13–0.83, *P* = 0.019), lower MoCA score (OR = 0.89; 95% CI: 0.82–0.97, *P* = 0.008), and fewer SN-EPVS (OR = 0.73; 95% CI = 0.53–1.00, *P* = 0.048) were independent risk factors for PD (Table 3). This model achieved an AUC of 0.57 (95% CI: 0.47–0.66).

**TABLE 3:** Logistic regression analysis for clinical and imaging predictors of PD.

**Variables**	**Univariate**		**Multivariable**	
	**OR (95% CI)**	***P*-value**	**OR (95% CI)**	***P*-value**
**Clinical variables**				
Age, years	0.97 (0.94–1.00)	0.068	–	–
Sex, male	0.55 (0.29–1.06)	0.075	0.33 (0.13–0.83)	**0.019**
Smoking	1.01 (0.46–2.19)	0.987	–	–
Hypertension	1.14 (0.56–2.32)	0.726	–	–
Hyperlipidaemia	3.51 (0.89–13.80)	0.072	–	–
Diabetes mellitus	0.71 (0.16–3.09)	0.649	–	–
MoCA	0.91 (0.85–0.98)	**0.008**	0.89 (0.82–0.97)	**0.008**
**Imaging findings**				
CSO-EPVS	0.97 (0.94–1.01)	0.102	–	–
BG-EPVS	0.94 (0.89–0.99)	**0.031**	–	–
SN-EPVS	0.71 (0.55–0.92)	**0.009**	0.73 (0.53–1.00)	**0.048**
BS-EPVS	0.86 (0.70–1.07)	0.172	–	–
WMH score	0.76 (0.60–0.95)	**0.017**	–	–

*OR, odds ratio; MoCA, Montreal Cognitive Assessment; EPVS, enlarged perivascular spaces; CSO, centrum semiovale; BG, basal ganglia; SN, substantia nigra; BS, brainstem; WMH, White matter hyperintensity. P-value < 0.05.*

### Correlation Analysis of EPVS With Clinical Characteristics

**FIGURE 3:**
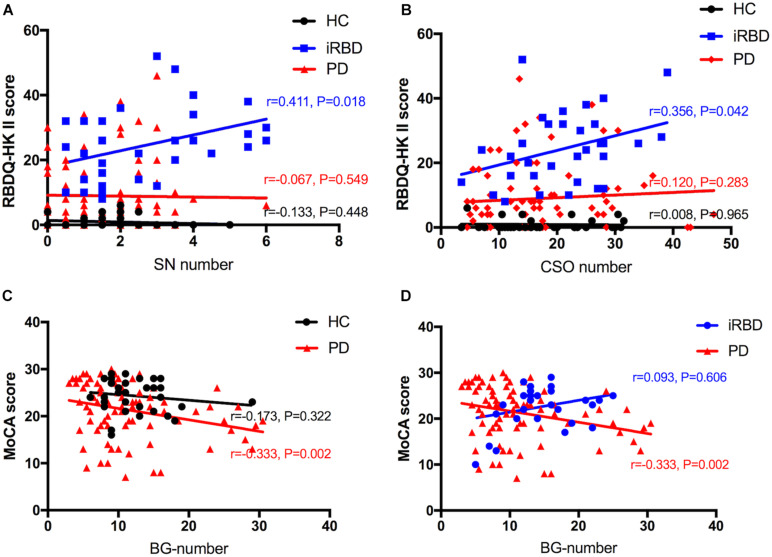
**A–D**: Correlation between number of EPVS and clinical features among HC, iRBD, and PD groups. HC, healthy control; iRBD, idiopathic rapid eye movement sleep behavior disorder; PD, Parkinson’s disease; EPVS, enlarged perivascular space; CSO, centrum semiovale; SN, substantia nigra; RBDQ-HK: Rapid Eye Movement Sleep Behavior Disorder Questionnaire-Hong Kong; MoCA: Montreal Cognitive Assessment.

The results of the correlation analysis of numbers of MRI-visible EPVS with clinical features in iRBD and PD patients are shown in Figure 3. Spearman’s rank correlation showed that numbers of SN-EPVS and CSO-EPVS were positively correlated with RBDQ-HK total scores (*r* = 0.333, *P* = 0.058; and *r* = 0.334, *P* = 0.057, respectively) and RBDQ-HK II scores (*r* = 0.411, *P* = 0.018; and *r* = 0.356, *P* = 0.042, respectively) in iRBD group, but not in HC and PD groups (*P* > 0.05) (Figures 3A,B). Spearman’s rank correlation also showed that the number of BG-EPVS was negatively correlated with MoCA score (*r* = −0.333, *P* = 0.002) in PD group, but not in HC and iRBD groups (*P* > 0.05) (Figures 3C,D). There was no correlation between MRI-visible EPVS numbers with UPDRS (total and III) score or H-Y stage in PD group (*P* > 0.05).

## Discussion

In this study, we assessed the clinical significance of MRI-visible EPVS in patients with iRBD and PD. Our study indicated that iRBD patients had significantly greater EPVS burdens (CSO, BG, SN, and BS) than PD patients. Moreover, iRBD patients had significantly greater EPVS burdens (CSO, BG) than PD without RBD, and significantly greater EPVS burdens (BG, SN, and BS) than PD with RBD. Higher CSO-EPVS and BS-EPVS burdens might be independent risk factors for iRBD, and higher CSO-EPVS and SN-EPVS burdens were positively correlated with the severity of clinical symptom in iRBD patients. Furthermore, higher BG-EPVS burden was positively correlated with the severity of cognitive impairment in PD patients.

Parkinson’s disease is characterized by the excessive aggregation of α-synuclein (Dehay et al., 2015). A range of mechanisms are involved in the pathogenesis of synucleinopathies, including oxidative stress, neuroinflammation and mitochondrial dysfunction (Blandini, 2013; Xu et al., 2019; Yang et al., 2020). Among them, inflammation has been recognized as the key factor (Wang et al., 2020; Zheng et al., 2020). Kishimoto et al. (2019) revealed that gut microbiota changes were associated with intestinal inflammation, which may contribute to α-synuclein misfolding (Zheng et al., 2020). By measuring serum superoxide dismutase (SOD) with lipoprotein cholesterol and CRP, respectively, in PD patients, (Yang et al., 2020) found the lower level of SOD along with cholesterol, HDL-C and LDL-C, and higher C-reactive protein (CRP) levels were correlated with the severity of PD. iRBD is considered to be associated with synucleinopathies, such as PD, dementia with Lewy bodies (DLB), and multiple system atrophy (MSA), and usually precedes the onset of first motor symptom of these diseases (Reichmann, 2017; Zhang et al., 2020). In iRBD patients, increased microglial activation was detected by positron emission tomography in the SN, implying that neuroinflammation may have occur in the early pathophysiological stage of α-synucleinopathies, including the prodromal phase of PD (Stokholm et al., 2017; Zhang et al., 2020).

Recently, the term “glymphatic” has been applied to the PVS system, based on its similarities to the lymphatic system in other tissue (Weller et al., 2009; Jessen et al., 2015). This waste drainage system can transport soluble proteins – such as amyloid beta (Aβ), whose deposition is thought to be the main pathogenic factor in AD – from the brain interstitial fluid (ISF) into the PVS system (Banerjee et al., 2017). However, the PVS system of fluid drainage and waste clearance is not fully understood. PVS burden has been associated with brain atrophy with aging, inflammation leading to increased oxygen consumption, cerebrovascular reactivity and hypoperfusion, and disruption of the blood-brain barrier (Joutel et al., 2010; Aribisala et al., 2014; Adams et al., 2015; Rost et al., 2018; Ghali et al., 2020). Recent findings suggest that the PVS system may be an immunological hotspot, which is the site of antigen presentation and tissue-resident memory T-cell reactivation (Smolders et al., 2020). Moreover, higher level of inflammation markers (CRP, interleukin-6, et al.) are associated with MRI-visible EPVS burdens, but not with WMH (Aribisala et al., 2014; Hilal et al., 2018a; Yang et al., 2020). These evidence link inflammation to the PVS system, as its malfunction may impair the clearance of α-synuclein aggregation, and causes subsequent progression of iRBD and PD. Therefore, we propose that MRI-visible EPVS burden may indicate obstruction by protein and stagnation of fluid (Brown et al., 2018), which serves as an imaging marker of neurodegenerative diseases, including PD and iRBD.

Studies on the relationship between EPVS and clinical characteristics of PD are limited. There was no significant difference in numbers of EPVS between PD (with and without RBD) and HC groups in the present study. We consider the following explanations for this observation. First, EPVS, which is a type of cerebral SVD, may play the same role as cerebrovascular factors that influence some aspects of the symptoms of PD. Second, dopamine has been shown to increase excitotoxic ischemic damage in experimental models, whereas dopamine antagonists ameliorate such effects, suggesting that decreased dopamine may be a protective factor for cerebrovascular disease (Lotharius et al., 1999). In addition, smoking is a confirmed protective factor for PD, yet it is associated with increased risk of stroke.

Parkinson’s disease patients frequently suffer from mild cognitive impairment (MCI) (MoCA score more than 21 and less than 26) (Litvan et al., 2012), who are at higher risk of developing dementia compared to PD patients with normal cognition (PD-NC) (Kehagia et al., 2010). Several lines of evidence suggested the inflammatory risk factors (Trefoil Factor 3, neutrophils, lymphocytes, et al.) in PD may modulate underlying neurodegeneration particularly in relation to dementia (Stojkovic et al., 2018; Zou et al., 2018; Nicoletti et al., 2020; Wang et al., 2020). It is promising to detect MRI-visible EPVS burden as a marker of cognitive decline in PD. Park Y. W. et al. (2019) suggested that PD-MCI subgroup had a higher BG-PVS burden than PD-NC subgroup. In our cohort, MoCA scores were low in all four groups. However, several Chinese validation studies of the application of MoCA to identify patients with dementia showed lower thresholds than those found in international populations, due to the differences in regional culture and level of education, especially in older patients (Zheng et al., 2012; Huang et al., 2018). The MoCA scores in our study were similar to the data in the above Chinese studies. We found the BG-EPVS burden was positively correlated with the severity of cognitive impairment in PD, which is consistent with previous studies. Recently, (Jia et al., 2019) found PD-MCI patients exclusively exhibited atrophy in the right entorhinal cortex compared to PD-NC by examining structural MRI. Further studies may investigate in the correlation between MRI-visible EPVSs and other neuroanatomical states in the cognitive aspect of PD.

There was no significant difference of EPVS burdens between PD patients with RBD and those without RBD in our cohort. We consider the following explanations for this observation. First, MRI-visible EPVSs, which may indicate diminished glymphatic clearance of α-synuclein (Jessen et al., 2015; Zou et al., 2019), were potentially related to the clinical events in PD (Laitinen et al., 2000). However, there was no significant difference in demographic or clinical characteristics in these two groups. Second, although iRBD shows a high rate of conversion to PD, only a minority of newly diagnosed PD cases show RBD (Sixel-Doring et al., 2014), and the sequence of the occurrence of RBD and the typical clinical symptoms of PD are diverse, which may be the result of individual differences, the exposure environment, and other factors. Therefore, neuropathological changes in the brain in PD with RBD phenotype are not yet conclusive, and further animal and human studies are required to elucidate this process (Schenck et al., 2013; Tekriwal et al., 2017).

Intriguingly, it is documented that the glymphatic system drains ISF and metabolic waste via PVSs more effectively during sleep (Xie et al., 2013). Therefore, it is plausible that sleep-related disorders disrupt waste clearance mechanisms via PVS system and potentially contribute to the accumulation of toxic proteins such as beta amyloid (Aβ), τ-protein, and α-synuclein. Quite recently, (Del Brutto et al., 2019) suggested that poor sleep efficiency was independently associated with BG-EPVSs. Sleep involves transitions between three different states: wakefulness, REM sleep, and non-REM sleep. RBD is characterized by loss of paralysis during REM sleep, allowing patients to act out vivid, often unpleasant dreams (Schenck et al., 2013). Therefore, it is conceivable that sleep-related disorders in iRBD may alter these clearance mechanisms, thus favoring a higher EPVS burden. Most insights into RBD have been drawn from animal lesion studies and functional models, which show that REM sleep regulation predominantly involved the key pontine centers, with additional modulation by the SN, BG, and frontal cortex (St Louis and Boeve, 2017). According to the Braak’s hypothesis, RBD emerges when α-synuclein pathology affects brainstem areas that are involved in sleep regulation (Braak et al., 2003). Our study revealed a relationship between iRBD and higher EPVS burdens in CSO, BG, SN, and BS. Moreover, CSO-EPVS and SN-EPVS may be positively correlated with the severity of clinical symptoms in iRBD. This is the first clinical study to specifically report such clinical associations.

Contrary to previous theories that MRI-visible EPVS indicates poor waste clearance and predicts worse clinical outcomes (Abbott et al., 2018), we found iRBD patients had significantly higher EPVS burdens (CSO, BG, SN, and BS) than PD patients. Recently, a study found a significantly lower MRI-visible EPVS volume in the anterosuperior medial temporal lobe in some patients with early cognitive decline (Sepehrband et al., 2020). This suggested impaired signaling due to MRI-visible EPVS might be a manifestation of severe brain waste drainage dysfunction, which is consistent with our result.

In iRBD patients, sleep disruption during REM and neuroinflammation may lead to the increased metabolity deposition and α-synuclein aggregation, thus favoring a high EPVS burden. As the disease progresses, poor PVS drainage may cause increased deposition of metabolites and α-synuclein aggregation, and in a vicious cycle, increased α-synuclein aggregation may obstruct the PVS drainage system further, leading to lower MRI-visible EPVS burdens in PD. However, the underlying mechanism requires further verification. Finally, it is not clear whether lower EPVS burdens result from PD progression and impaired clearance of fluid, or conversely, that impaired fluid clearance occurs as a result of EPVS. However, our results demonstrated that, as a prodromal marker of PD, patients with iRBD obviously had higher EPVS burdens than patients with PD, either with or without RBD symptoms. We therefore speculate that EPVS burden may play a compensatory role in the process of iRBD developing into PD, and as the disease progresses, adaptation may become decompensatory. Further research based on this mechanism is needed to elucidate this process.

### Limitations

This study has several limitations. First, there is limited sample size, and future research with a larger sample size is required to examine the reproducibility of the current findings in other cohorts. Second, according to the second edition of the International Classification of Sleep Disorders, polysomnography is essential to establish the criterion for the diagnosis of RBD, however, it is costly, labor-intensive, and impractical to perform in large study populations. Some of the iRBD patients underwent polysomnography, and others were tested with RBDQ-HK scale in this study. RBDQ-HK is a self-administered questionnaire, and its scores (total and factor 2) are useful and validated RBD screening instruments. It has also been demonstrated to have satisfactory reliability and validity as a tool for screening for the severity of iRBD, especially in east China (Shen et al., 2014). Third, we calculated EPVS numbers manually, and this is time-consuming and prone to subjectivity. Automated, computational quantification methods may be necessary for assessing EPVS in the future. However, as our analysis methods are simple with good inter-rater reliability, they can be applied easily in clinical practice. Finally, this is a neuroimaging study that only reflects the different EPVS patterns in iRBD and PD patients in a cross-sectional study. To investigate how iRBD patients transit to PD over time and demonstrate an underlying mechanism for this transition, we will follow-up these patients for a longitudinal cohort study to make the conclusions more solid in the future.

## Conclusion

Our study provides evidence that iRBD and PD patients have different MRI-visible EPVS burdens. iRBD patients have significantly higher EPVS burdens than PD patients, an interesting observation that requires further evaluation, as it may be relevant to the mechanism of glymphatic system clearance caused by α-synuclein aggregation. Further research based on a comprehensive consideration of EPVS burden and the association between MRI-visible EPVS and α-synuclein aggregation is necessary to clarify the mechanisms underlying these findings.

## Data Availability Statement

The raw data supporting the conclusions of this article will be made available by the authors, without undue reservation.

## Ethics Statement

The study involving human participants was reviewed and approved by the ethics committee of the Second Affiliated Hospital of the Zhejiang University School of Medicine, and written consent was obtained from each participant. The patients/participants provided their written i nformed consent to participate in this study. Written informed consent was obtained from the individual(s) for the publication of any potentially identifiable images or data included in this article.

## Author Contributions

XS: conceptualization, data curation, formal analysis, validation, and writing original draft. LG: conceptualization, data curation, validation, and writing original draft. ZS and CZ: data curation, validation, and writing original draft. CJ, JW, and TG: investigation and data curation. TG and YF: investigation and formal analysis. XG and XX: methodology and Formal analysis. XY and YY: investigation, methodology, review and editing. MZ: funding acquisition and resources. JP: project administration, resources, supervision, review and editing. All the authors read and approved the final manuscript.

## Conflict of Interest

The authors declare that the research was conducted in the absence of any commercial or financial relationships that could be construed as a potential conflict of interest.
